# Prospective study evaluating the relative sensitivity of ^18^F-NaF PET/CT for detecting skeletal metastases from renal cell carcinoma in comparison to multidetector CT and ^99m^Tc-MDP bone scintigraphy, using an adaptive trial design

**DOI:** 10.1093/annonc/mdv289

**Published:** 2015-07-22

**Authors:** E. L. Gerety, E. M. Lawrence, J. Wason, H. Yan, S. Hilborne, J. Buscombe, H. K. Cheow, A. S. Shaw, N. Bird, K. Fife, S. Heard, D. J. Lomas, A. Matakidou, D. Soloviev, T. Eisen, F. A. Gallagher

**Affiliations:** 1 Department of Radiology, Addenbrooke's Hospital, Cambridge University Hospitals NHS Foundation Trust and Cambridge University Health Partners, Cambridge; 2 Department of Radiology, University of Cambridge, Cambridge; 3 MRC Biostatistics Unit Hub for Trials Methodology, Cambridge; 4 Department of Nuclear Medicine, Addenbrooke's Hospital, Cambridge University Hospitals NHS Foundation Trust and Cambridge University Health Partners, Cambridge; 5 East Anglian Regional Radiation Protection Service, Cambridge University Hospitals NHS Foundation Trust and Cambridge University Health Partners, Cambridge; 6 Department of Oncology, Addenbrooke's Hospital, Cambridge University Hospitals NHS Foundation Trust and Cambridge University Health Partners, Cambridge; 7 Cancer Research UK Cambridge Institute, University of Cambridge, Cambridge; 8 Department of Oncology, University of Cambridge, Cambridge, UK

**Keywords:** renal cell carcinoma, bone metastases, ^18^F-NaF PET/CT, ^99m^Tc-MDP bone scintigraphy, computed tomography

## Abstract

**Background:**

The detection of occult bone metastases is a key factor in determining the management of patients with renal cell carcinoma (RCC), especially when curative surgery is considered. This prospective study assessed the sensitivity of ^18^F-labelled sodium fluoride in conjunction with positron emission tomography/computed tomography (^18^F-NaF PET/CT) for detecting RCC bone metastases, compared with conventional imaging by bone scintigraphy or CT.

**Patients and methods:**

An adaptive two-stage trial design was utilized, which was stopped after the first stage due to statistical efficacy. Ten patients with stage IV RCC and bone metastases were imaged with ^18^F-NaF PET/CT and ^99m^Tc-labelled methylene diphosphonate (^99m^Tc-MDP) bone scintigraphy including pelvic single photon emission computed tomography (SPECT). Images were reported independently by experienced radiologists and nuclear medicine physicians using a 5-point scoring system.

**Results:**

Seventy-seven lesions were diagnosed as malignant: 100% were identified by ^18^F-NaF PET/CT, 46% by CT and 29% by bone scintigraphy/SPECT. Standard-of-care imaging with CT and bone scintigraphy identified 65% of the metastases reported by ^18^F-NaF PET/CT. On an individual patient basis, ^18^F-NaF PET/CT detected more RCC metastases than ^99m^Tc-MDP bone scintigraphy/SPECT or CT alone (*P* = 0.007). The metabolic volumes, mean and maximum standardized uptake values (SUV_mean_ and SUV_max_) of the malignant lesions were significantly greater than those of the benign lesions (*P* < 0.001).

**Conclusions:**

^18^F-NaF PET/CT is significantly more sensitive at detecting RCC skeletal metastases than conventional bone scintigraphy or CT. The detection of occult bone metastases could greatly alter patient management, particularly in the context when standard-of-care imaging is negative for skeletal metastases.

## Abstract (precis)

Current imaging techniques for detecting bone metastases in renal carcinoma are insensitive to small lesions. This prospective trial of ^18^F-NaF PET/CT shows it is more than twice as sensitive as CT and more than three times as sensitive as bone scintigraphy for these lesions. ^18^F-NaF PET/CT could alter management by identifying occult metastases in patients with negative standard-of-care imaging.

## introduction

Renal cell carcinoma (RCC) is the 13th most common cancer worldwide with an estimated 63 920 new cases in the United States in 2014 and patients may have advanced or unresectable disease at presentation [1]. Approximately a third of patients with RCC develop bone metastases and these may cause significant morbidity including pain, loss of mobility, spinal cord compression, hypercalcaemia and pathological fractures [2].

Thirty percent of patients treated by nephrectomy with curative intent for localized RCC develop metastatic disease on follow-up, which may result from occult metastases that were present at the time of surgery [2]. Therefore, optimized preoperative detection and localization of metastases is important for guiding management. A sensitive test for bone metastases could help avoid unnecessary surgery in patients with metastatic disease, or allow solitary lesions to be surgically resected when additional metastases are confidently excluded [3]. In addition, recent advances in the medical management of patients with advanced RCC require companion imaging biomarkers to accurately assess disease and monitor drug efficacy [4].

Current practice for imaging patients with RCC is contrast-enhanced computed tomography (CT) of the thorax, abdomen and pelvis in combination with ^99m^Tc-labelled methylene diphosphonate (^99m^Tc-MDP) skeletal scintigraphy, if there are features to suggest bone metastases. Since bone lesions from RCC are often lytic with a poor osteoblastic response, there may be limited uptake of the ^99m^Tc-MDP tracer. Multidetector CT has been reported to have a sensitivity of only 66% for vertebral metastases and a recent meta-analysis found a sensitivity of only 59% for the detection of bone metastases with bone scintigraphy across a range of cancers [5, 6]. More sensitive tests for detecting bone metastases are therefore required.

^18^Fluorine-labelled sodium fluoride (^18^F-NaF) is a highly sensitive positron emission tomography (PET) tracer for detection of increased bone turnover, which occurs within the metastases of many tumors [7, 8]. ^18^F-NaF was originally used as an agent for skeletal scintigraphy with a standard gamma camera, before it was superseded by ^99m^Tc-MDP. However, with the improved availability of PET technology, ^18^F-NaF has re-emerged as an agent for skeletal imaging [7, 8], and the combination of ^18^F-NaF PET imaging with CT enables accurate anatomical localization of ^18^F uptake. Despite the promising role of ^18^F-NaF PET/CT in other cancers, there are limited data demonstrating the diagnostic performance of the technique for RCC metastases [9, 10]. We present a prospective study of patients with known RCC bone metastases, comparing the sensitivity of ^18^F-NaF PET/CT to ^99m^Tc-MDP bone scintigraphy [including pelvic single photon emission computed tomography (SPECT)] and CT alone. The primary aim was to identify whether ^18^F-NaF PET/CT has increased sensitivity for the detection of occult bone metastases compared with current standard-of-care imaging and consequently whether it may have a role in the routine management of patients with the disease.

## patients and methods

The trial was undertaken with ethical and institutional approval as a Clinical Trial of an Investigative Medicinal Product under the Medicines and Healthcare Products Regulatory Agency. Patients with metastatic (stage IV), pathologically confirmed RCC were recruited from the renal oncology clinic between May 2012 and August 2013. All tumor subtypes were included. Patients either had confirmed bone metastases (diagnosed by standard-of-care bone scintigraphy or CT) or suspected bone metastases (with bone pain, bone mass or neurological symptoms likely due to bone metastases).

### imaging

^99m^Tc-MDP bone scintigraphy was carried out 3 h after i.v. administration of 600 MBq ^99m^Tc-MDP. Anterior and posterior whole-body planar bone scintigrams were acquired from vertex to toes using a Discovery NM630 SPECT or 670 SPECT/CT system (GE Healthcare) fitted with high-resolution, low-energy collimators. Images were acquired over 15 min. SPECT imaging of the pelvis was carried out over 25 min using a 128 × 128 matrix and a 50 × 40 cm field of view. SPECT images were reconstructed and displayed as transverse and orthogonal slices. The pelvis was selected for additional imaging given the difficulty of discriminating overlapping structures in this region on planar imaging.

^18^F-NaF PET/CT imaging of the whole body was carried out 60 min after i.v. administration of 250 MBq ^18^F-NaF using a Discovery 690 PET/CT system (GE Healthcare). The ^18^F-NaF was manufactured by the Wolfson Brain Imaging Centre, University of Cambridge, as an Investigational Medicinal Product. An initial unenhanced high-resolution CT was carried out from vertex to toes, which was subsequently used for attenuation correction of the PET data. CT images were acquired at 120 kV with variable mA and a slice thickness of 3.75 mm and reconstructed using soft tissue and bone algorithms. PET imaging was carried out with a total of 12–15 bed positions (3 min/position). The PET images were fused with the CT images on an Advantage workstation (GE Healthcare) and displayed individually and together as transverse and orthogonal slices.

### reporting

Each imaging modality was reported independently by a single radiologist/nuclear medicine physician with ≥5 years’ experience in reporting as an attending/consultant, using a routine clinical workstation (GE Healthcare). Each lesion was reported with a malignancy certainty score from 1 to 5 (supplementary Table S1, available at *Annals of Oncology* online). Discriminating features included increased/decreased tracer uptake, location, pattern, size and aggressive appearance.

Lesions identified on ^99m^Tc-MDP bone scintigraphy or SPECT were recorded as having either low/high tracer uptake. Lesions identified by CT were reported as lytic (low attenuation) or sclerotic (high attenuation). Lesions identified by ^18^F-NaF PET/CT were recorded as having low or high tracer uptake, or as only visible on the CT element of the PET/CT. The time to report each study was also recorded.

Given the dual nature of PET/CT, lesions reported as malignant by the radiologist (scoring 4–5) were reviewed by a nuclear medicine physician who also gave a certainty score for malignancy and these scores were then averaged.

The initial imaging reports were used for the primary analysis. Following this, the reporters discussed the lesions with discrepancies between the modalities. Lesions associated with surgical interventions were excluded due to difficulty in discriminating a benign response to intervention, from residual or recurrent malignant disease. Certainty scores were adjusted for some lesions following multimodality team discussion, as would occur in the setting of a tumor board or multidisciplinary team meeting. Reports were adjusted to include all lesions identified by each modality. The secondary analysis was carried out on these consensus data. Follow-up imaging was reviewed by searching the hospital image archive for subsequent standard-of-care imaging.

The lesions with ^18^F-NaF uptake were quantitatively analyzed using proprietary software (Advantage Workstation, GE Healthcare). A threshold of 75% of the SUV_max_ was used to calculate the SUV_mean_ and the metabolic volume defined by this threshold was recorded.

### statistics

The trial was powered to answer two primary research questions: whether ^18^F-NaF PET/CT detected more lesions than bone scintigraphy or CT alone, using the median number of lesions identified with each technique. A multiarm multistage design was used: 10 patients in the first stage and 10 in the second if necessary [11]. The futility and efficacy boundaries in the first stage were *P* values above 0.15 and below 0.05, respectively. For each hypothesis, the trial design had a 90% power with a 5% one-sided type I error rate where PET/CT provides an increase in the mean number of lesions detected from 2 to 3.5. As recommended for exploratory trials, we did not correct for the two primary hypotheses [12]. The maximum chance of making a type I error was 10%.

Lesions with a certainty score of 4–5 were considered malignant. Any lesion reported as malignant by one or more modality was presumed to represent a metastasis. To test the two primary research questions, a one-sided Wilcoxon-signed rank test was used (www.r-project.org). A Poisson generalized mixed-effects regression was used as a secondary analysis to test the difference in the mean number of lesions found; fixed effects for the modality used and random effects for each individual were included.

For analysis of the metabolic volume, SUV_mean_ and SUV_max_ of the benign and malignant lesions, a linear mixed-effects model was fitted with a random effect for each individual. In each case, the outcome variable was transformed to the log-scale to ensure homoscedasticity. The model parameters were used to derive *P* values testing the difference in mean metabolic volume, SUV_mean_ and SUV_max_ between malignant and benign lesions.

## results

Eleven patients were enrolled in the trial, but one withdrew due to discomfort during the ^18^F-NaF PET/CT imaging, leaving a total of 10 participants (Table 1). Follow-up imaging was reviewed for up to 2 years after the study imaging, with a minimum of 2 months (due to patient death). Three patients died during the trial.

**Table 1.** Patient characteristics

Characteristics	Number
Male	6
Female	4
Age range (years)	48–79
ECOG score 0	4
ECOG score 1	3
ECOG score 2	3
Histology	6 clear cell
	1 papillary
	3 unclassified, poorly differentiated
Prestudy radiotherapy	3
Prestudy antiangiogenic agent (pazopanib, everolimus, sunitinib)	4
Preference for imaging modality	4 bone scintigraphy
	2 PET/CT
	4 no preference
Mean dose of ^99m^Tc-MDP	548.9 MBq (range 511–574)
Mean dose of ^18^F-NaF	253.5 MBq (range 234–274)
Mean time between ^99m^Tc-MDP bone scintigraphy/SPECT +^18^F-NaF PET/CT	8 days (range 3–17)

ECOG, Eastern Cooperative Oncology Group score.

### imaging outcomes

Based on the initial reports, ^18^F-NaF PET/CT detected more RCC metastases than ^99m^Tc-MDP bone scintigraphy/SPECT or CT alone on an individual patient basis (*P* = 0.0099 and 0.0098, respectively). Since these *P* values were below a value of 0.05, the trial was terminated early for efficacy.

**Figure 1.**
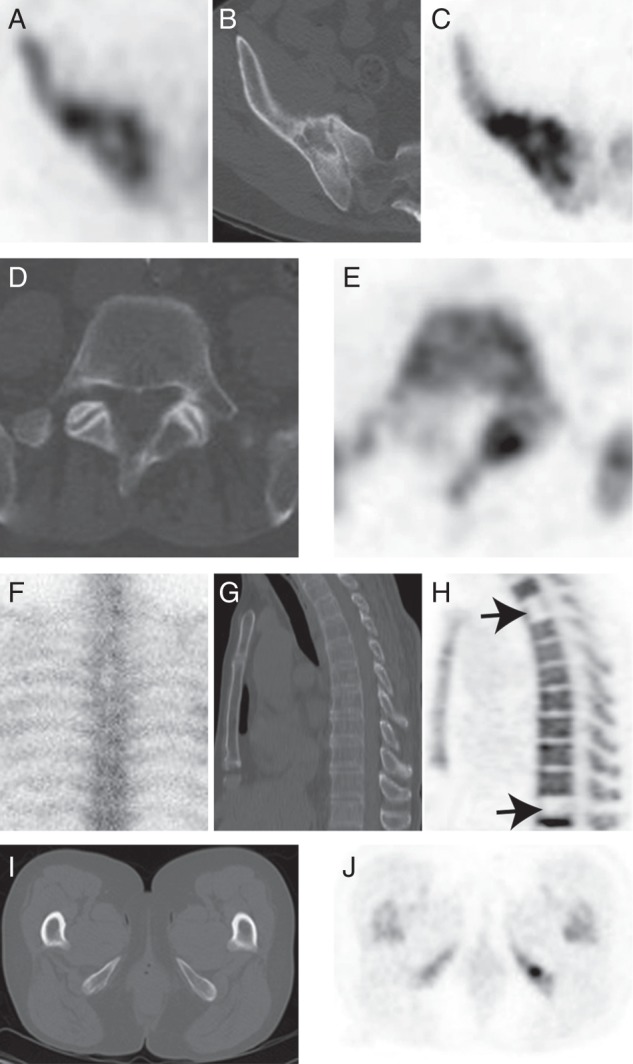
(A–C) Lesion in the right ilium detected by all three modalities: (A) axial ^99m^Tc-MDP SPECT; (B) axial CT; (C) axial ^18^F-NaF PET. (D and E) Example of ^18^F-NaF PET signal emphasizing a CT abnormality in the left posterior elements of the L5 vertebral body: (D) axial CT; (E) ^18^F-NaF PET. (F–J) Examples of lesions only identified on ^18^F-NaF PET/CT. Vertebral metastases: (F) ^99m^Tc-MDP bone scintigraphy coronal image. (G) CT sagittal reconstruction. (H) ^18^F-NaF PET sagittal reconstruction showing metastases with low tracer uptake (arrows). Metastasis in the inferior pubic ramus: (I) axial CT image; (J) ^18^F-NaF PET image.

Following multimodality review, a total of 77 lesions were diagnosed as malignant (Figure 1 and supplementary Table S2, available at *Annals of Oncology* online). 77/77 of these were identified by ^18^F-NaF PET/CT [100%; 95% confidence intervals (CI) 95.2% to 100%], 35/77 by CT alone (45.5%; 95% CI 34.8% to 56.5%) and 22/77 by ^99m^Tc-MDP bone scintigraphy/SPECT (28.6%; 95% CI 19.7% to 39.5%). The sensitivity of ^18^F-NaF PET/CT was more than double that of CT alone and three times more than that of ^99m^Tc-MDP bone scintigraphy/SPECT. CT and bone scintigraphy together, which reflects standard-of-care imaging for patients with suspected bone metastases, identified 50/77 of the metastases reported by ^18^F-NaF PET/CT (65%; 95% CI 53.8% to 74.7%).

On an individual patient basis, ^18^F-NaF PET/CT also detected more RCC metastases compared with ^99m^Tc-MDP bone scintigraphy/SPECT or CT alone (*P* = 0.007). There was no significant difference in the mean number of lesions found between CT and ^99m^Tc-MDP bone scintigraphy (incident risk ratio 1.591; 95% CI 0.940–2.691; *P* = 0.084); however, ^18^F-NaF PET/CT found significantly more lesions than ^99m^Tc-MDP bone scintigraphy (incident risk ratio 3.500; 95% CI 2.194–5.584; *P* < 0.001) and CT alone (incident risk ratio 2.200; 95% CI 1.484–3.262; *P* < 0.001). ^18^F-NaF PET/CT detected more metastases compared with the other modalities irrespective of patient performance status [Eastern Cooperative Oncology Group scores (ECOG); Table 1].

The average time to report the imaging was 14.5 min (range 10–20 min) for ^99m^Tc-MDP bone scintigraphy/SPECT, 11.0 min (range 9–17 min) for CT and 20.0 min (range 8–40 min) for ^18^F-NaF PET/CT.

### discrepancies and follow-up imaging

All 10 patients had follow-up CT imaging which was used to evaluate discrepancies between imaging modalities. Twenty-three lesions were only identified on ^18^F-NaF PET/CT (Figure 1F–J); 19 of these lesions were subsequently identified as malignant lesions on standard-of-care bone scintigraphy [6], CT [8] or magnetic resonance imaging (MRI) [5]. Three of the remaining lesions were in the extremities and were not included on follow-up imaging. The fourth lesion, in the femoral head, was stable on follow-up imaging and could represent a treated metastasis or a benign lesion.

For 10/77 lesions, there was intermodality disagreement on the malignant/benign nature of the lesions and a consensus could not be reached even after multimodality review (supplementary Table S3, available at *Annals of Oncology* online). Of the 10 discrepancies, 3 lesions were in the pelvis, 2 in vertebral bodies, 2 in the skull and 3 in the long bones. Seven of the 10 lesions were imaged on follow-up, and all were diagnosed as malignant. In addition, one metastasis was found on follow-up imaging that had not been reported by the trial ^18^F-NaF PET/CT: a patient developed a pathological clavicle fracture that, in retrospect, could be identified on the trial ^18^F-NaF PET/CT and had been missed at the time of reporting. Although this study was not designed to assess the true sensitivity, the relative sensitivities revised to reflect this extra metastasis are as follows: ^18^F-NaF PET/CT: 98.7% (95% CI 93.1% to 99.9%); CT alone: 44.9% (95% CI 34.3% to 55.9%) and ^99m^Tc-MDP bone scintigraphy/SPECT: 28.2% (95% CI 19.4% to 39.0%).

### lesion characteristics

The majority of metastases were lytic and showed high tracer uptake (supplementary Table S2, available at *Annals of Oncology* online). Sclerotic metastases were found in three patients imaged following treatment. Seven of the 77 lesions reported on ^18^F-NaF PET/CT were only identified on the CT component.

Quantitative analysis was carried out on 35 benign lesions (score 1–2) and 66 malignant lesions (score 4–5); some lesions could not be analyzed quantitatively due to their small size or location. Malignant lesions were significantly larger than benign lesions (*P* < 0.001): the median volume of the malignant lesions was 0.6 cm^3^ (range 0.1–5.1 cm^3^) and of the benign lesions was 0.3 cm^3^ (range 0.1–2.8 cm^3^). The mean difference in volume (log-scale) between benign and malignant lesions was 0.71 (95% CI 0.36 to 1.06; *P* < 0.001). The malignant lesions also showed a significantly higher SUV_max_ and SUV_mean_ (*P* < 0.001): the median SUV_max_ for the malignant lesions was 12.4 (range 4.4–38.3) compared with 10.1 for the benign lesions (range 2.5–25.3); the median SUV_mean_ for the malignant lesions was 11 (range 3.8–31.5) compared with 8.4 for the benign lesions (range 2.2–21.2). The mean difference in SUV_max_ (log-scale) was 0.54 (95% CI 0.32 to 0.76; *P* < 0.001). The mean difference in SUV_mean_ (log-scale) was 0.55 (95% CI 0.33 to 0.77; *P* < 0.001).

## discussion

Previous studies comparing bone scintigraphy, ^18^F-NaF PET/CT, ^18^F-FDG PET/CT and MRI for the detection of skeletal metastases have found ^18^F-NaF PET/CT to be superior for the detection of these metastases across a broad range of cancers [9, 10, 13, 14]. This work represents the first prospective study of the role of ^18^F-NaF PET/CT in metastatic RCC in conjunction with an adaptive trial design.

^18^F-NaF PET/CT demonstrated a significant number of metastases that were occult on standard-of-care imaging. The technique is more than twice as sensitive at detecting RCC bone metastases compared with CT alone and more than three times as sensitive compared with ^99m^Tc-MDP bone scintigraphy with pelvic SPECT. Collectively, CT and bone scintigraphy only identified 65% of the metastases identified by ^18^F-NaF PET/CT. The increased sensitivity of ^18^F-NaF PET/CT could be used to detect occult metastases in patients considered to have no bone metastases with current standard-of-care imaging if identification of these metastases would significantly alter patient management. These results represent the first step in addressing whether the technique can be used to select patients with resectable metastatic disease not identified by other imaging techniques, or patients with significant occult bone metastases which renders them unsuitable for surgery. The technique could also be used as an imaging biomarker for drug development and response to therapy in the nonoperative and neoadjuvant settings. However, this trial was powered only to identify the relative sensitivity of ^18^F-NaF PET/CT in detecting bone metastases in a population with known metastases. Other potential roles of the technique may be investigated in large multicenter trials in the future.

A limitation of this study is the lack of a gold standard for true metastases. The specificity of ^18^F-NaF PET/CT could not be determined as it was not feasible to verify all the lesions by biopsy due to the large number of lesions and the complexity of PET-guided biopsy. However, subsequent standard-of-care imaging detected 19 of the 23 lesions that were only detected by ^18^F-NaF PET/CT in the trial setting, showing that these lesions were correctly identified as malignant. Only four lesions were not verified as metastases on follow-up imaging; three lesions were outside of the imaged field and the fourth stable lesion could represent a treated metastasis or a benign lesion. No false positives were identified. Only one missed metastasis was found on follow-up imaging, which represented a reporting error rather than a true limitation of ^18^F-NaF PET/CT.

Intermodality disagreement was most common at sites with overlapping structures such as in the pelvis and vertebrae, leading to uncertainty as to whether small lesions are degenerative or malignant. In the skull, it can be difficult to differentiate metastases from normal variants, such as arachnoid granulations and venous lakes, or benign lesions. Quantitative analysis of the ^18^F-NaF PET data identified characteristics that may help to distinguish benign from malignant lesions where there is diagnostic uncertainty: the metabolic volume and tracer uptake of the malignant lesions was found to be significantly greater than that for the benign lesions. These features could be used in the clinical setting to aid diagnosis.

Although the trial was not specifically powered to address the relationship between the evaluation of bone metastases and ECOG score, it is interesting to note that ^18^F-NaF PET/CT detected more metastases even in patients with good performance status. While there have been previous studies evaluating the role of bone scintigraphy in metastatic renal patients with low ECOG scores [15], future trials addressing the role of ^18^F-NaF PET/CT in this group of patients are now required.

The adaptive study design proved successful in efficiently extracting the results from a small number of patients. Following a significant result after recruiting only 10 patients, the study was closed which avoided unnecessary further recruitment. The result emphasizes the potential role of well-designed adaptive studies in the context of imaging studies where high patient costs may be prohibitive for large trials.

There are two published case studies on the use of ^18^F-NaF PET/CT for the detection of RCC metastases [16, 17]. The first found that all the lytic lesions on CT demonstrated ^18^F-NaF uptake, with five additional fluoride-avid lesions that were not seen on CT [16]. The second found that ^18^F-NaF PET/CT detected three additional lesions compared with bone scintigraphy [17]. Our findings are supported by a recent pilot study which showed an increased detection rate of bone metastases with ^18^F-NaF PET/CT in 13 patients imaged with ^18^F-NaF PET/CT (from skull to thigh only) and bone scintigraphy [18]. Our prospective study compared whole-body ^18^F-NaF PET/CT to bone scintigraphy and CT alone in all patients and demonstrated the statistically significant superiority of ^18^F-NaF PET/CT over current routine imaging modalities.

Compared with ^99m^Tc-MDP, ^18^F-NaF has several advantages including rapid bone uptake and blood clearance, it can be imaged within an hour of i.v. administration, and it has an excellent signal-to-noise ratio [7, 8]. The radiation dose from 250 MBq of ^18^F-NaF (6 mSv) is similar to that from 600 MBq of ^99m^Tc-MDP (3 mSv). The high-resolution full-body CT component of the PET/CT has an associated dose of 16 mSv; as CT imaging is routinely carried out as part of standard-of-care imaging, this could be combined with the PET imaging for attenuation correction. Although the PET/CT took longer to report than the other two techniques, this was only 5 min more than that for bone scintigraphy and 9 min more than that for CT. On a practical level, there have been shortages of the precursor to ^99m^Tc, molybdenum-99, which makes alternative radionuclides increasingly important to maintain a clinical service for the detection of bone metastases [19].

Our study is the first prospective trial comparing ^18^F-NaF PET/CT imaging to ^99m^Tc-MDP bone scintigraphy and CT alone for detecting RCC bone metastases. ^18^F-NaF PET/CT was found to be superior at detecting metastases compared with the other techniques, and detected a number of metastases that were not identified by standard-of-care imaging. The detection of these occult metastases can significantly alter the management of patients who are diagnosed as being metastasis free with standard-of-care imaging, for example those being assessed for surgery with curative intent. Guidelines for the imaging of patients with RCC should consider inclusion of ^18^F-NaF PET/CT for bone metastases.

## funding

This work was supported by Cancer Research UK (grant number C19212/A16628) and the imaging was funded by the National Institute of Health Research Cambridge Biomedical Research Centre. The authors also received research support from the Engineering and Physical Sciences Research Council Imaging Centre in Cambridge and Manchester and the Cambridge Experimental Cancer Medicine Centre. The research has also been partly funded by a generous donation from the family and friends of a patient.

## disclosure

FG has research support from GE Healthcare. All remaining authors have declared no conflicts of interest.

## Supplementary Material

Supplementary Data
