# Supplementary material for: Prospective study evaluating the relative sensitivity of 18F-NaF PET/CT for detecting skeletal metastases from renal cell carcinoma in comparison to multidetector CT and 99mTc-MDP bone scintigraphy, using an adaptive trial design
Source: Ann Oncol. 2015 Jul 22;26(10):2113–8. doi: 10.1093/annonc/mdv289 (PMC4576907; doi:10.1093/annonc/mdv289)
Supplement: Supplementary Data [file supp_26_10_2113__index.html]

Prospective study evaluating the relative sensitivity of 18F-NaF PET/CT for detecting skeletal metastases from renal cell carcinoma in comparison to multidetector CT and 99mTc-MDP bone scintigraphy, using an adaptive trial design — Prospective study evaluating the relative sensitivity of 18F-NaF PET/CT for detecting skeletal metastases from renal cell carcinoma in comparison to multidetector CT and 99mTc-MDP bone scintigraphy, using an adaptive trial design — Prospective study evaluating the relative sensitivity of 18F-NaF PET/CT for detecting skeletal metastases from renal cell carcinoma in comparison to multidetector CT and 99mTc-MDP bone scintigraphy, using an adaptive trial design — Supplementary Data 

# Prospective study evaluating the relative sensitivity of 18F-NaF PET/CT for detecting skeletal metastases from renal cell carcinoma in comparison to multidetector CT and 99mTc-MDP bone scintigraphy, using an adaptive trial design

## Supplementary Data

Supplementary Data

- Supplementary Table 1 - docx file
- Supplementary Table 2 - docx file
- Supplementary Table 3 - docx file
