# Supplementary material for: Prospective study evaluating the relative sensitivity of 18F-NaF PET/CT for detecting skeletal metastases from renal cell carcinoma in comparison to multidetector CT and 99mTc-MDP bone scintigraphy, using an adaptive trial design
Source: Ann Oncol. 2015 Jul 22;26(10):2113–8. doi: 10.1093/annonc/mdv289 (PMC4576907; doi:10.1093/annonc/mdv289)
Supplement: Supplementary Data [file supp_mdv289_mdv289supp_table1.docx]

**S1. Scoring system for the lesions.**

| **Score** | **Definition** |
| --- | --- |
| 1 | Highly likely to be benign |
| 2 | Likely to be benign |
| 3 | Intermediate |
| 4 | Likely to be malignant |
| 5 | Highly likely to be malignant |
