# Supplementary material for: Prospective study evaluating the relative sensitivity of 18F-NaF PET/CT for detecting skeletal metastases from renal cell carcinoma in comparison to multidetector CT and 99mTc-MDP bone scintigraphy, using an adaptive trial design
Source: Ann Oncol. 2015 Jul 22;26(10):2113–8. doi: 10.1093/annonc/mdv289 (PMC4576907; doi:10.1093/annonc/mdv289)
Supplement: Supplementary Data [file supp_mdv289_mdv289supp_table2.docx]

**S2. Characteristics of lesions reported as metastases**

| Characteristic | Descriptor | Number of lesions |
| --- | --- | --- |
| Location | Axial skeleton  Appendicular skeleton | 61/77 (79.2%)  16/77 (20.8%) |
| CT | Lytic  Sclerotic  Mixed | 31/35 (88.6%)  3/35 (8.6%)  1/35 (2.8%) |
| ^99m^Tc-MDP bone scintigraphy/SPECT | High tracer uptake  Mixed high/low uptake  Low tracer uptake | 20/22 (90.9%)  2/22 (9.1%)  0/22 (0%) |
| **^18^**F-NaF PET/CT | High tracer uptake  Mixed high/low uptake  Low tracer uptake  CT only | 56/77 (72.7%)  7/77 (9.1%)  7/77 (9.1%)  7/77 (9.1%) |
