# Supplementary material for: Prospective study evaluating the relative sensitivity of 18F-NaF PET/CT for detecting skeletal metastases from renal cell carcinoma in comparison to multidetector CT and 99mTc-MDP bone scintigraphy, using an adaptive trial design
Source: Ann Oncol. 2015 Jul 22;26(10):2113–8. doi: 10.1093/annonc/mdv289 (PMC4576907; doi:10.1093/annonc/mdv289)
Supplement: Supplementary Data [file supp_mdv289_mdv289supp_table3.docx]

**S3. Malignant lesions detected by ^18^F-PET/CT and how they were reported by bone scintigraphy or CT alone.**

| **Reported as malignant by both modalities (*n* = 7)** | | |
| --- | --- | --- |
| **Bone scintigraphy** | **CT** | **Number of lesions** |
| Malignant | Malignant | 7 |
| **Reported as malignant by one modality (*n* = 43)** | | |
| **Bone scintigraphy** | **CT** | **Number of lesions** |
| Malignant | No lesion detected | 11 |
| No lesion detected | Malignant | 26 |
| Intermediate | Malignant | 2 |
| Malignant | Benign | 1 |
| Malignant | Intermediate | 3 |
| **Reported as non-malignant/not detected (*n* = 27)** | | |
| **Bone scintigraphy** | **CT** | **Number of lesions** |
| No lesion detected | Benign | 1 |
| No lesion detected | Intermediate | 2 |
| Benign | Intermediate | 1 |
| No lesion detected | No lesion detected | 23 |
